# Right-sided infective endocarditis or thrombus? Report of two cases diagnosed by transthoracic echocardiography

**DOI:** 10.1186/s13019-024-02522-3

**Published:** 2024-02-03

**Authors:** Wenjing Ge, Bijun Wu, Zhen Wang, Haichun Zhang

**Affiliations:** Department of Ultrasound, The Affiliated Guangdong Second Provincial General Hospital of Jinan University, Guangdong, China

**Keywords:** Right-sided infective endocarditis, thrombus, Echocardiography, Differential diagnosis

## Abstract

**Background:**

Right-sided infective endocarditis (RSIE) is a relatively uncommon condition which is difficult to distinguish from thrombus, particularly when the site of infection or the patient’s symptoms are atypical. There have been few reports exploring the differential diagnostic and analytical features between RSIE and thrombus.

**Case presentation:**

Here, we presented two cases of RSIE—one involving the tricuspid valve and the other affecting the pulmonary artery. Notably, the second case was initially misdiagnosed as thrombus based on the findings of by computed tomography angiography(CTA).

**Conclusions:**

Vegetation and thrombus can be distinguished according to the nature of the mass, its attachment location, and the clinical manifestation. Echocardiography can observe both the location and size of the mass, and the dynamic changes in cardiac hemodynamics and cardiac morphology, thereby facilitating an effective distinction between vegetation and thrombus.

**Supplementary Information:**

The online version contains supplementary material available at 10.1186/s13019-024-02522-3.

## Background

Infective endocarditis (IE) is an infection resulting from the migration of pathogenic microorganisms to the heart valve or ventricular endocardium. It is relatively uncommon in childhood. However, it can be life-threatening [1]. Despite an increased incidence of IE due to improved survival rates among children with congenital heart disease (CHD), left-sided IE remains more prevalent compared to right-sided infective endocarditis (RSIE) [1]. Existing literature has described the etiology, clinical manifestations, ultrasonic features, and treatments for RSIE [2], but few delve into the detailed differential diagnosis between RSIE and thrombus. In this paper, we present two cases of RSIE, one of which was initially misdiagnosed as thrombosis through computed tomography angiography (CTA). Our objective was to analyze the differential diagnosis of RSIE and thrombus while emphasizing the importance of diagnostic reasoning in ultrasound-based diagnoses of RSIE, thereby cultivating correct diagnostic thinking and reducing the rate of misdiagnosis.

## Case presentation

### Case1

A 6-year-old girl was admitted to our hospital with a persistent fever and cough lasting two weeks. She had a preexisting ventricular septal defect (VSD) since birth. Physical examination revealed an elevated body temperature of 101.3 degrees F, tachycardia with a heart rate of 110 beats per minute, and a holosystolic murmur detected between the third and fourth intercostal spaces on the left edge of the sternum. Laboratory results showed an increased white blood cell (WBC) count of 21.8 × 10^9^/L (normal range: 5.0–12.0 × 10^9^/L), predominantly neutrophils at 83% (normal range: 40–69%), low hemoglobin levels at 64 g/L (normal range: 105–145 g/L), and significantly elevated C-reactive protein (CRP) levels at 100.7 mg/L (normal range: 0.0–8.0 mg/L). Chest x-ray revealed focal pulmonary infiltrates in both lower lung fields. Three consecutive sets of blood cultures were collected and were negative at 24 h, after which two additional sets of blood cultures were added, and after 48 h all blood cultures remained negative.

**Fig. 1:**
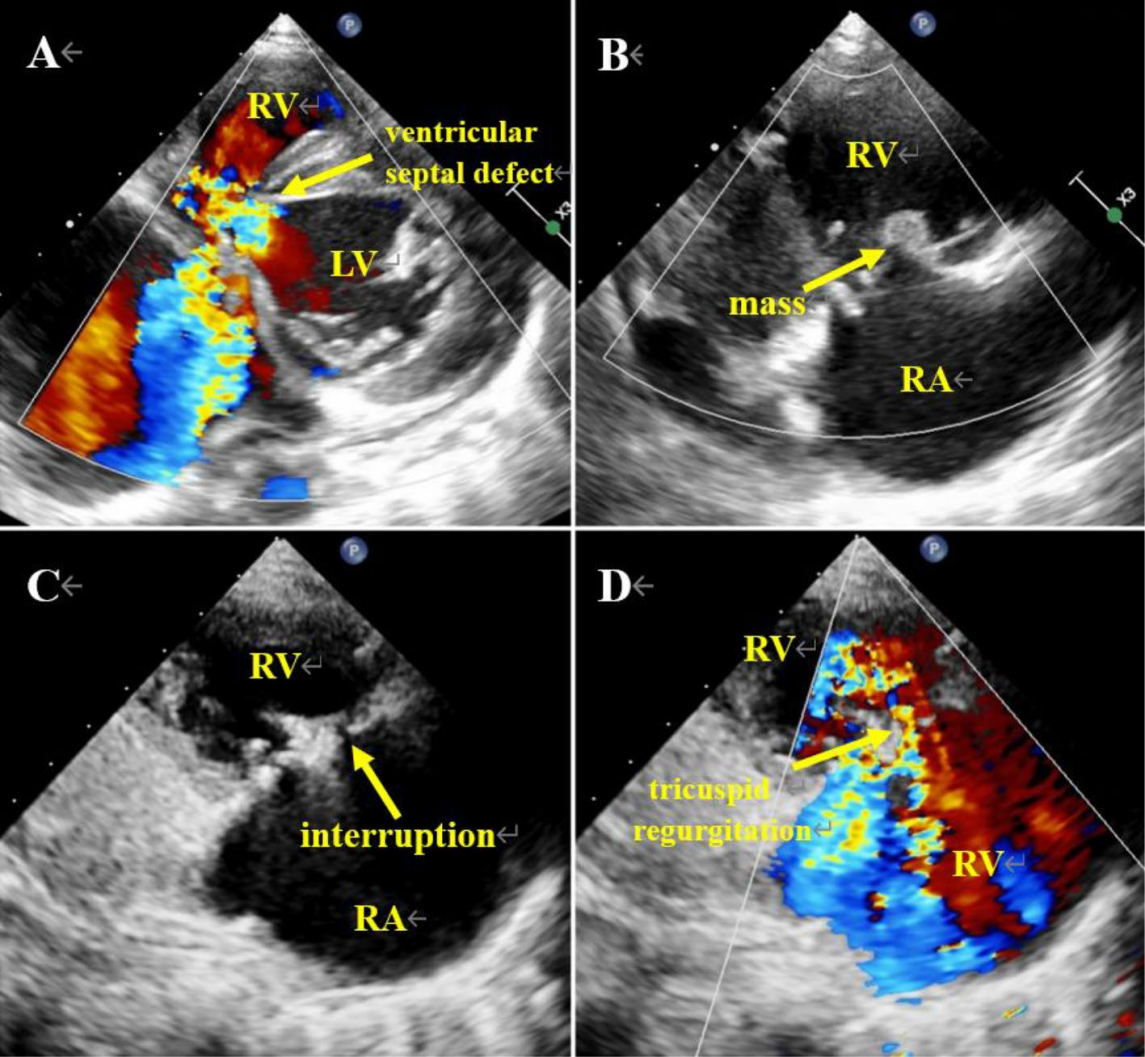
Echocardiography images of Case 1. (**A**) Color doppler flow imaging (CDFI) shows a ventricular septal defect (VSD) of 0.5 cm (arrow), shunting from left to right. (**B**) Tricuspid anterior leaflet atrial surface is slightly hyperechoic with a mass of about 0.9 × 0.6 cm (arrow), oscillating with the anterior leaflet. (**C**) Arrow shows interrupted echo continuity of the anterior lobe of the tricuspid valve (about 0.2 cm). (**D**) The CDFI shows tricuspid regurgitation, in which a beam of regurgitation comes from the interrupted echo of the anterior lobe of the tricuspid valve (arrow). LV = left ventricle; RV = right ventricle; RA = right atrium

Transthoracic echocardiography (TTE) demonstrated overall cardiomegaly and a perimembranous VSD measuring 0.4 cm, resulting in a left-to-right shunt (Fig. 1A, Additional file 1: Video 1, Additional file 2: Video 2). Additionally, a slightly hyperechoic mass measuring 1.1 × 0.5 cm was observed attached to the right atrial surface of the anterior tricuspid valve. The continuity of the anterior tricuspid valve was disrupted, leading to severe tricuspid regurgitation during systole, characterized by regurgitat flow passing through the interruption of the anterior tricuspid valve (Fig. 1B-D, Additional file 3: Video 3, Additional file 4: Video 4).

Several considerations support the diagnosis of IE rather than thrombosis in this case. Firstly, the presence of a VSD puts the patient at risk for IE, and the disruption of the tricuspid valve may be a complication associated with IE. Secondly, the girl exhibited symptoms of high fever, elevated WBC count, and increased CRP levels, indicating an infection. The administration of antibiotics prior to blood culture may have affected the reliability of the results. Lastly, the absence of other medical conditions, combined with her active lifestyle, made thrombosis highly unlikely. Considering these factors, the observed mass was strongly suspected to be a vegetation rather than a thrombus. Subsequent detection of bacterial DNA in surgically removed tissue confirmed the diagnosis of IE, with *Streptococcus pseudopneumoniae* identified as the causative pathogen.

### Case 2

A 15-year-old girl was initially diagnosed with CHD, specifically severe pulmonary stenosis and mild tricuspid regurgitation, at our hospital two years ago. However, she did not receive treatment at that time and subsequently experienced chest tightness, dyspnea, and bilateral limb edema, resulting in prolonged bed rest. Presently, she was admitted to the hospital due to heart failure, classified as New York Heart Association (NYHA) Class III. On physical examination, anasarca was observed, accompanied by slightly cyanotic lips. Cardiac dullness extended to the left, while a rapid heart rate, diminished heart sounds, and a systolic ejection murmur in the second intercostal space of the left margin of the sternum were detected. Laboratory findings indicated underlying infections, coagulation abnormalities, heart failure, and hypoalbuminemia. Specifically, the WBC count was elevated at 27.8 × 10^9^/L (normal range: 5.0–12.0 × 10^9^/L), predominantly consisted by neutrophils at 81% (normal range: 40–69%). CRP levels were significantly increased at 34.3 mg/L (normal range: 0.0–8.0 mg/L), platelet count was low at 90.0 × 10^9^/L (normal range: 140–440 × 10^9^/L), D-dimer levels were elevated at 12.4 µg/ml (normal range: 0-0.5 µg/ml), N-terminal pro-brain natriuretic peptide levels were markedly elevated at 9846 pg/ml (normal range: <125 pg/ml), and albumin levels were reduced at 22.9 g/L (normal range: 40–50 g/L). Chest X-ray revealed multiple patchy and cloudy high-density lesions scattered throughout both lung fields, as well as bilateral pleural effusion. Pericardial effusion smears and 5 blood cultures for 48 h did not reveal the presence of fungi, bacteria, or acid-fast bacilli.

**Fig. 2:**
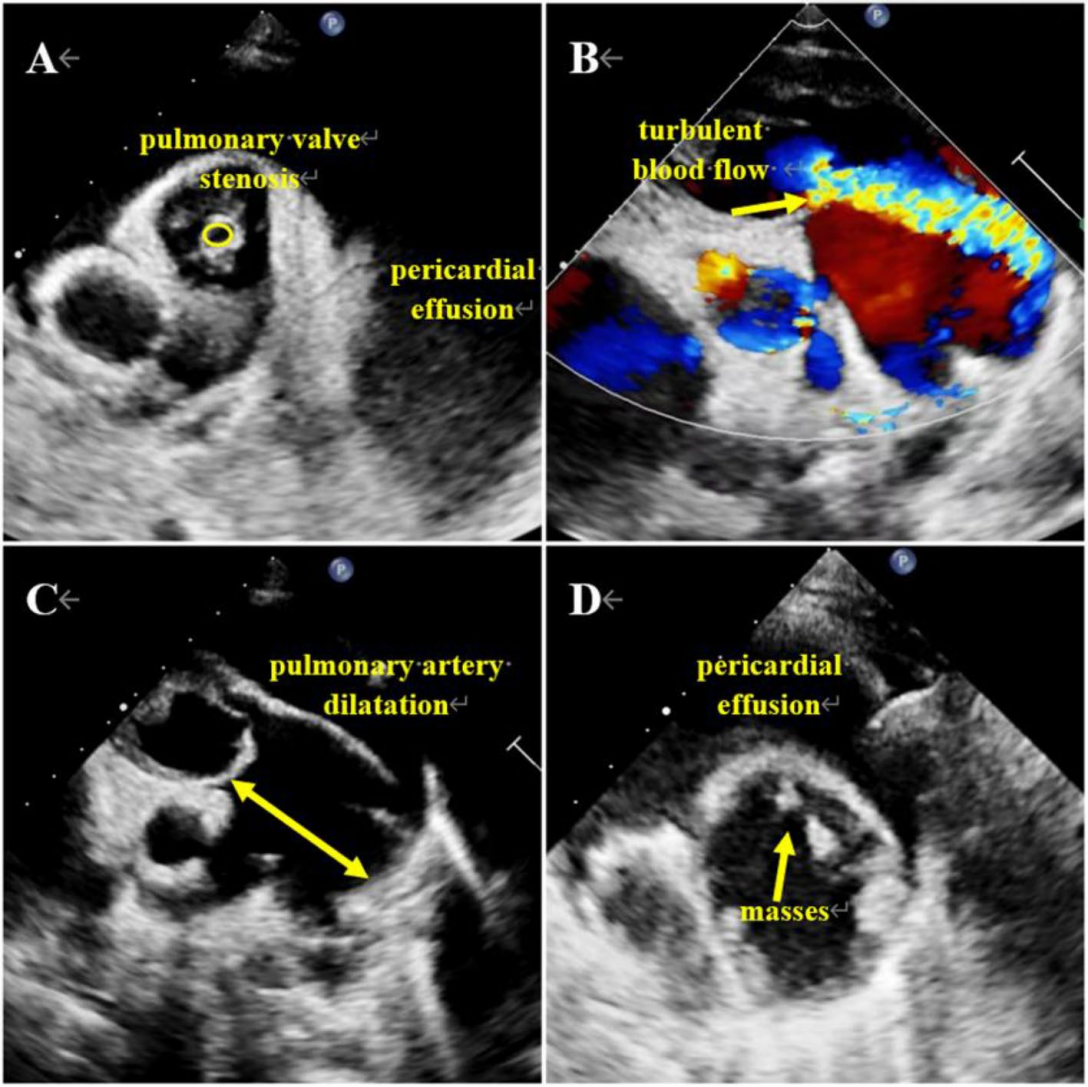
Echocardiography images of Case 2. (**A**) Pericardial effusion and pulmonary valve stenosis with an open area of 0.12 cm2 (oval). (**B**) Color doppler flow imaging shows that the turbulent blood flow signal in the pulmonary artery is brightly colored (arrow). (**C**) Pulmonary artery dilatation and trunk width of 4 cm (arrow). (**D**) Several masses in the dilated pulmonary artery (arrow)

TTE demonstrated massive pericardial effusion, right artrioventricular enlargement, and ventricular thickening (Additional file 5: Video 5). Additionally, the pulmonary valve leaflets were observed to be elongated and thickened, restricting the opening of the pulmonary valve (Fig. 2A, Additional file 6: Video 6). A bunch of turbulent blood flow was observed from the pulmonary valve into the main pulmonary artery during systole (Fig. 2B, Additional file 7: Video 7), with a peak flow velocity of approximately 5.6 m/s. TTE also revealed significant dilatation of the main pulmonary artery, measuring approximately 4 cm in width (Fig. 2C), accompanied by the presence of multiple masses attached to the arterial wall (Fig. 2D, Additional file 8: Video 8).

**Fig. 3:**
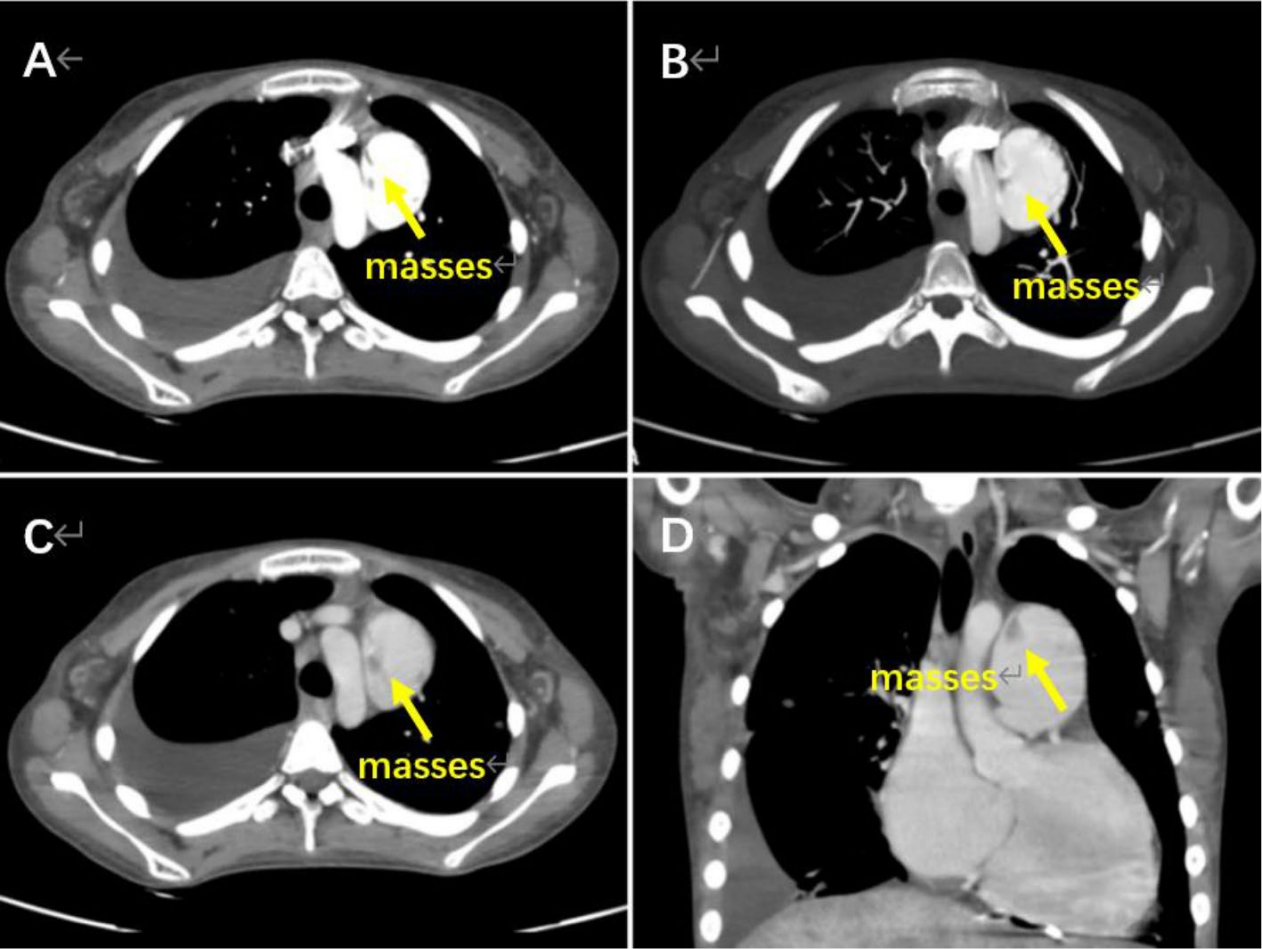
CTA images of case 2. Several masses in the dilated pulmonary artery (arrow)

Therefore, we identified the masses as vegetations due to several factors: (1) The patient had a history of CHD and pulmonary artery stenosis, which increased the risk of pulmonary artery infective endartitis; (2) The masses in this patient were located near turbulent blood flow, whereas thrombosis tends to occur in areas with slow blood flow; (3) The girl’s chest X-ray showed pulmonary infiltrates, and laboratory data indicated an elevated WBC count and CRP levels, suggesting that she was infected. However, the patient’s CTA suggested that the masses were thrombi (Fig. 3A-D, Additional file 9: Video 9). Consequently, she underwent extracorporeal cardiac surgery. Subsequently, *Streptococcus pseudopneumoniae* was identified as the causative bacteria of the pulmonary artery infective endartitis.

## Discussion and conclusions

The risk factors for RSIE include CHD, pacemaker use, and indwelling deep venous catheterization (particularly associated with intravenous drug dependence). Among these factors, CHD is the most common predisposing condition for RSIE, with ventricular septal defects and patent ductus arteriosus being the most frequently observed anatomical abnormalities. The right heart vegetation and its fragments can detach and enter the pulmonary circulatory system, potentially leading to complications such as pulmonary embolism, pneumonia, or lung abscess. In addition to persistent fever, RSIE patients often present with respiratory symptoms including chest pain, dyspnea, cough, and hemoptysis. Hypertrophic vegetations are typically found on the tricuspid valve and endocardium, along with a larger size (≥ 20 mm). It could be attributed to the lower pressure within the right heart cavity, which is beneficial to plant growth. The conventional imaging method to detect IE is TTE, but transesophageal echocardiography (TEE) is more sensitive. In addition, cardiac computed tomography, fluorine-18 fluorodeoxyglucose positron emission tomography/computed tomography, and single photon emission computed tomography/computed tomography with isotope labelling of leukocytes can be used as imaging tests for IE. Staphylococcus aureus and Streptococcus viridis are the common pathogens associated with RSIE. Empirically, penicillin (with β-lactamase inhibitor) can be used to combat infection in RSIE patients with severe underlying complications, while a combination of third-generation cephalosporins and fluoroquinolones may be used in RSIE patients with underlying cardiac structural abnormalities [3]. However, surgical intervention is recommended for patients with right heart failure who do not respond effectively to medical treatment, as well as the patients with recurrent bacterial pulmonary embolism, moderate to severe tricuspid regurgitation, or right heart failure that remain uncontrolled despite medical management [4].

IE is characterized by insidious onset, variable symptoms, rapid progression, and high mortality rates (> 30%) [5], making its diagnosis and treatment challenging and often resulting in underdiagnosis, delayed diagnosis, or misdiagnosis. Echocardiography plays a crucial role in identifying the location, size, and shape of vegetations, as well as assessing structural cardiac changes and associated hemodynamic alterations [6]. Understanding the mechanisms underlying vegetation formation is key to its diagnosis. Three conditions for bacterial IE were as follows: (1) presence of valve thrombus, bacteria in the circulation; (2) Bacterial growth on the valve [7]; (3) High-velocity blood flow. They can damage to the contact surfaces, leading to thrombosis and promoting vegetation development.

Compared to left-sided IE, RSIE typically manifests as fevers of unknown origin, bacteremia, and other infection symptoms and complications resulting from damage to the right heart [8]. In the first case, the patient’s symptoms, laboratory data, and X-rays indicated an infection, combined with echocardiographic evidence of VSD (the prerequisite) and perforated lobular regurgitation (complications), enabling a relatively straightforward diagnosis of vegetation. Conversely, in the second case, differentiating between infective vegetation and thrombus was more challenging. On the one hand, the patient had CHD, abnormal laboratory findings of elevated WBC count and CRP, as well as inflammatory changes observed on X-ray, suggesting the possibility of infective endartitis. However, multiple blood cultures yielded negative results. On the other hand, the patient exhibited anasarca, prolonged bed rest, and abnormal blood coagulation function, indicating a potential thrombotic event. These indications were contradictiory in nature.

Several aspects can help differentiate between thrombus and vegetation. Firstly, the nature of the mass is different: thrombi have a smooth surface, while infectious vegetations are more fragile with uneven internal echoes due to variations in formation times. Old thrombi adhere closely to the vascular endothelium and do not easily detach, while infectious vegetations are prone to rupture and embolize, leading to infarction and abscess formation. Secondly, clinical manifestations are also different: thrombi are often associated with high-risk factors for thrombosis, such as atrial fibrillation and myocardial infarction, while infective vegetation presents with typical signs and symptoms of IE, including positive blood cultures. In the presented cases, the negative blood culture results in both patients may have been influenced by the administration of antibiotics before blood collection. Lastly, the attachment site provides further clues: thrombi tend to form in areas with slow blood flow, such as the left atrial appendage and apex, where they remain relatively stationary. In contrast, vegetations typically adhere to sites with turbulent blood flow and move in synchrony with blood flow or valve opening and closing. Infectious vegetation can also cause erosive damage, such as valvular ulcer perforation and valve annulus abscesses. For example, in the second case, the wall of the pulmonary artery was impacted by turbulent blood flow. Given the rapid blood flow, thrombus formation becomes challenging, and even if formed, it quickly dislodges due to the high-speed blood flow.

In conclusion, the location of IE can vary depending on the underlying CHDs, necessitating careful consideration during diagnosis. When echocardiography reveals a mass without typical signs, a comprehensive evaluation incorporating patient history, clinical manifestations, and biochemical indexes is essential. Furthermore, since IE often leads to multiple infections and vegetation formation, ultrasound examinations should be meticulous, thorough, and comprehensive.

### Electronic supplementary material

Below is the link to the electronic supplementary material.

Supplementary Material 1: Additional file 1 Video 1.mp4: A perimembranous ventricular septal defect (arrow)

Supplementary Material 2: Additional file 2 Video 2.mp4: Color doppler flow imaging of a perimembranous ventricular septal defect (arrow)

Supplementary Material 3: Additional file 3 Video 3.mp4: A mass attached to the anterior tricuspid valve (arrow)

Supplementary Material 4: Additional file 4 Video 4.mp4: Massive tricuspid regurgitations and a beam of regurgitation flow passing through the interruption of the anterior leaflet of the tricuspid valve (arrow)

Supplementary Material 5: Additional file 5 Video 5.mp4: Massive pericardial effusion, right artrioventricular enlargement, and ventricular thickening (arrow)

Supplementary Material 6: Additional file 6 Video 6.mp4: The pulmonary valve leaflets are long and thick, which limits the opening of the pulmonary (arrow)

Supplementary Material 7: Additional file 7 Video 7.mp4: A turbulent blood flows from the pulmonary valve during systole to the left wall of the main pulmonary artery (arrow)

Supplementary Material 8: Additional file 8 Video 8.mp4: Multiple masses attached nearby the left wall of the main pulmonary artery (arrow)

Supplementary Material 9: Additional file 9 Video 9.mp4: The movies of computed tomography angiography show masses in the dilated pulmonary artery (arrow)

## Data Availability

Records and data pertaining to this case are in the patient’s secure medical records at the Affiliated Guangdong Second Provincial General Hospital of Jinan University. All data generated or analysed during this study are included in this published article and its supplementary information files.

## Abbreviations

RSIE: Right-sided infective endocarditis
CTA: Computed tomography angiography
IE: Infective endocarditis
CHD: Congenital heart disease
VSD: Ventricular septal defect
WBC: White blood cell
CRP: C-reactive protein
TTE: Transthoracic echocardiography
NYHA: New York Heart Association
TEE: transesophageal echocardiography
